# Genome‐wide association of Tau imaging and plasma biomarkers in adults with Down Syndrome

**DOI:** 10.1002/alz.092615

**Published:** 2025-01-09

**Authors:** Kang‐Hsien Fan, Muhammad Muaaz Aslam, Lam‐Ha T Dang, Priyanka Gorijala, Ruyu Shi, Eleanor Feingold, M. Ilyas Kamboh, Joseph H. Lee, Laura Xicota, Ann D Cohen, Benjamin L Handen, Bradley T. Christian, Elizabeth Head, Mark Mapstone, Carlos Cruchaga, Alzheimer’s Biomarker Consortium – Down Syndrome (ABC‐DS)

**Affiliations:** ^1^ School of Public Health, University of Pittsburgh, Pittsburgh, PA USA; ^2^ Columbia University Irving Medical Center, New York, NY USA; ^3^ Washington University School of Medicine, Saint Louis, MO USA; ^4^ Department of Human Genetics, University of Pittsburgh, Pittsburgh, PA USA; ^5^ Taub Institute for Research on Alzheimer’s Disease and the Aging Brain, Columbia University, New York, NY USA; ^6^ Gertrude H Sergievsky Center, Columbia University Irving Medical Center, New York, NY USA; ^7^ University of Pittsburgh Alzheimer's Disease Research Center (ADRC), Pittsburgh, PA USA; ^8^ University of Pittsburgh, Pittsburgh, PA USA; ^9^ Department of Medical Physics, University of Wisconsin‐Madison School of Medicine and Public Health, Madison, WI USA; ^10^ University of California, Irvine, Irvine, CA USA; ^11^ NeuroGenomics and Informatics Center, Washington University School of Medicine, St Louis, MO USA

## Abstract

**Background:**

Adults with Down Syndrome (DS) clearly show a higher risk of developing Alzheimer's disease (AD) when compared with the general population. Thus, it is important to investigate the role AD‐related biomarkers in adults with DS. Here we have performed genome‐wide association analyses on Tau‐PET and plasma Tau levels in the participants of the Alzheimer’s Biomarker Consortium – Down Syndrome (ABC‐DS).

**Method:**

Genome‐wide analyses were performed on 320 individuals of European descent DS (ranging in age from 25 to 81, Male 54.1%) having biomarker data from Tau‐PET as well as plasma p‐tau 181, p‐tau 217, and total tau. The association of these biomarkers with two APOE SNPs, rs429358 (E4) and rs7412 (E2) was also examined. We used a joint model of multiple phenotypes to investigate the genetic factors contributing to all Tau biomarkers simultaneously in those participants having complete phenotypic and genotypic data.

**Result:**

In the APOE association analysis, APOE4 was not significantly associated with any biomarker. However, individuals carrying APOE2 had lower plasma p‐tau‐181 levels (p= 0.0017) than non‐carriers. In a genome‐wide analysis, we identified three novel genome‐wide significant (GWS) associations. The first association was with plasma total Tau on chromosome 11 near JHY (top SNP= rs77264104; MAF=0.017; p=1.75E‐08; β=‐1.52). The second association was with Tau‐PET on trisomy chromosome 21 near a long coding RNA LOC105372747 (top SNP= rs76523946; MAF=0.034; p=2.04E‐08; β=0.61). Lastly, the multiple phenotype analysis revealed association with all Tau AD biomarkers on chromosome 7 in the intronic region of SEM1 (Top SNP= rs78223947; MAF= 0.039; p=1.44E‐08).

**Conclusion:**

We have identified three novel associations with Tau biomarkers in adults with DS associated with either lower tau or higher tau biomarkers. Two of these associations (JHY and SEM1) have also been implicated potentially with Tau pathology and AD risk, respectively, in non‐DS participants. This investigation underscores the potential of leveraging the DS population as a valuable resource for discerning genetic elements that contribute to AD or AD‐related biomarkers.

